# Case Report: Successful concomitant surgical interventions for dilated ascending aorta and aortic valve insufficiency in a severe haemophilia A case

**DOI:** 10.3389/fmed.2026.1824001

**Published:** 2026-05-21

**Authors:** Cristina Emilia Ursu, Margit Serban, Ciprian Tomuleasa, Adina Traila, Dan Cioata, Raluca Staicu, Delia Savescu, Teodora Smaranda Arghirescu

**Affiliations:** 1 Romanian Academy of Medical Sciences, Onco-Haematology Research Unit, Children's Emergency Hospital “Louis Turcanu” Timisoara, European Haemophilia Treatment Centre, Timișoara, Timiș, Romania; 2 Department of Haematology, Research Centre for Functional Genomics and Translational Medicine, Iuliu Hațieganu University of Medicine and Pharmacy, Cluj Napoca, Romania; 3 Medical Centre for Evaluation Therapy, Medical Education and Rehabilitation of Children and Young Adults, European Haemophilia Comprehensive Care Centre, Buzias, Romania; 4 Cardiovascular Surgery Clinic and Anaesthesia and Intensive Care Clinic, Institute of Cardiovascular Diseases, Timisoara, Romania; 5 Laboratory Department, Children's Emergency Hospital “Louis Turcanu” Timisoara, European Haemophilia Treatment Centre, Timișoara, Timiș, Romania; 6 Department of Paediatrics, Division of Onco-Haematology, “Victor Babes” University of Medicine and Pharmacy Timișoara, Timișoara, Romania

**Keywords:** aortic valve insufficiency, balancing haemostatic factors, cardiac surgery, dilated ascending aorta, severe haemophilia A

## Abstract

**Introduction:**

Cardiac surgery is the most challenging major invasive procedure in haemophilia. It involves the use of coagulation factor replacement, heparin therapy, antifibrinolytics, platelet antiaggregants, and is associated with surgical trauma, extracorporeal circulation, cardioplegia, and hypothermia, making it highly complex.

**Method:**

Case presentation.

**Results:**

We present our experience managing a 64-year-old patient with severe haemophilia A (SHA) without inhibitors who underwent open aortic valve replacement and reduction plasty of the dilated ascending aorta under cardiopulmonary bypass (CPB). His history included SHA complications and cardiac failure due to severe aortic regurgitation. In accordance with international guidelines, the following have been decided: surgical intervention, complex medical therapy, a rigorous multidisciplinary team approach, and accurate, proper laboratory monitoring. The biological valve was successfully implanted, and the aorta was tubularly reduced to a 35 mm diameter, while maintaining its symmetry. The patient was cared for in the intensive care unit for 2 days, with CPB weaned after 1 h and 20 min; he was discharged after 10 days, with a recommendation for continuous prophylaxis with FVIII, combined with fractionated heparin for 3 months, and thereafter switched to aspirin for another 3 months. Immediate and long-term outcomes were good, with no bleeding, thrombotic events, or other complications.

**Discussion/conclusion:**

Balancing conflicting hemostatic factors in a controversial biological environment was a challenging life-saving task. It required accurate, continuous lab monitoring as a key prerequisite for personalised therapy. The immediate and long-term evolution was good, free of complications.

## Introduction

Remarkable advances in the diagnosis and therapy of haemophilia have been documented over the past decades; safer, newer generations of coagulation factors and non-factor products have been developed, extending life expectancy and improving the quality of life for persons with haemophilia (PwH) to levels comparable with those of the non-haemophilic population. Consequently, haemophilia increasingly presents age-related morbidities, such as ischaemic heart disease and degenerative heart valve conditions, and the frequency of cardiac surgeries is steadily rising (1–5).

Undoubtedly, cardiac surgery is regarded as the most complex and intricate major invasive procedure for PwH. This highly demanding operation is associated with at least three categories of risk factors: those related to the bleeding disorder itself, the demanding surgical procedure and those related to medication. A main objective is to balance opposing effects, some thrombogenic and others bleeding-favouring, and to achieve a safe haemostatic level in practice. This is obtained through a mandatory multidisciplinary team approach, with high expertise in invasive procedures (interventional cardiologist, intensive care specialist, haematologist and laboratory expert), supported by competent, accurate laboratory monitoring, which is indispensable for a secure perioperative haemostatic condition and key to the success of open-heart surgery with cardiopulmonary bypass (CPB) (6–8).

Multiple publications worldwide have documented case reports and series on this life-saving, but also complex and risky procedure, specifically open-heart surgery with CPB, supporting our attempt in a patient with severe haemophilia A (9–11).

All the challenges associated with the surgical intervention are presented and discussed in our subsequent case report, along with medical therapy and haemostasis management.

## Case report

We present a 64-year-old man with severe haemophilia A (FVIII < 1%), without inhibitors, with recurrent haemarthroses leading to chronic haemophilic arthropathy, managed with on-demand or short-term prophylaxis using factor VIII concentrate, supported by physiotherapy. Also known to have chronic hepatitis C and essential arterial hypertension. The genetic test was not performed, and he has no family history of sudden cardiac death or congenital heart disease. He presented with worsening congestive heart failure symptoms, including dyspnea, fatigue, and palpitations. On exam, a decrescendo diastolic murmur was heard at the left sternal border, with signs of chronic arterial insufficiency like widened pulse pressure, additionally with jugular venous distension, and peripheral edema. He referred for management of cardiac failure due to severe aortic valve insufficiency. On echocardiographic examination, dilation of the ascending aorta (50 mm in diameter) was observed, along with left ventricular dilatation, an ejection fraction of 50%, and mitral and tricuspid regurgitation. The indication for surgery was based on accepted guidelines that apply equally to PwH and to those without this condition (12). The patient was well integrated into our haemophilia treatment centre and received strong support, but he expressed anxiety about the risks of open-heart surgery due to his bleeding disorder.

The technically demanding procedure was supported by medical therapy, including preoperative, intraoperative, and postoperative factor VIII plasma-derived (pdFVIII) replacement. In accordance with international guidelines, 5,000 IU of pd. FVIII concentrate was administered 45 to 50 min before the surgical incision.

**Figure 1:**
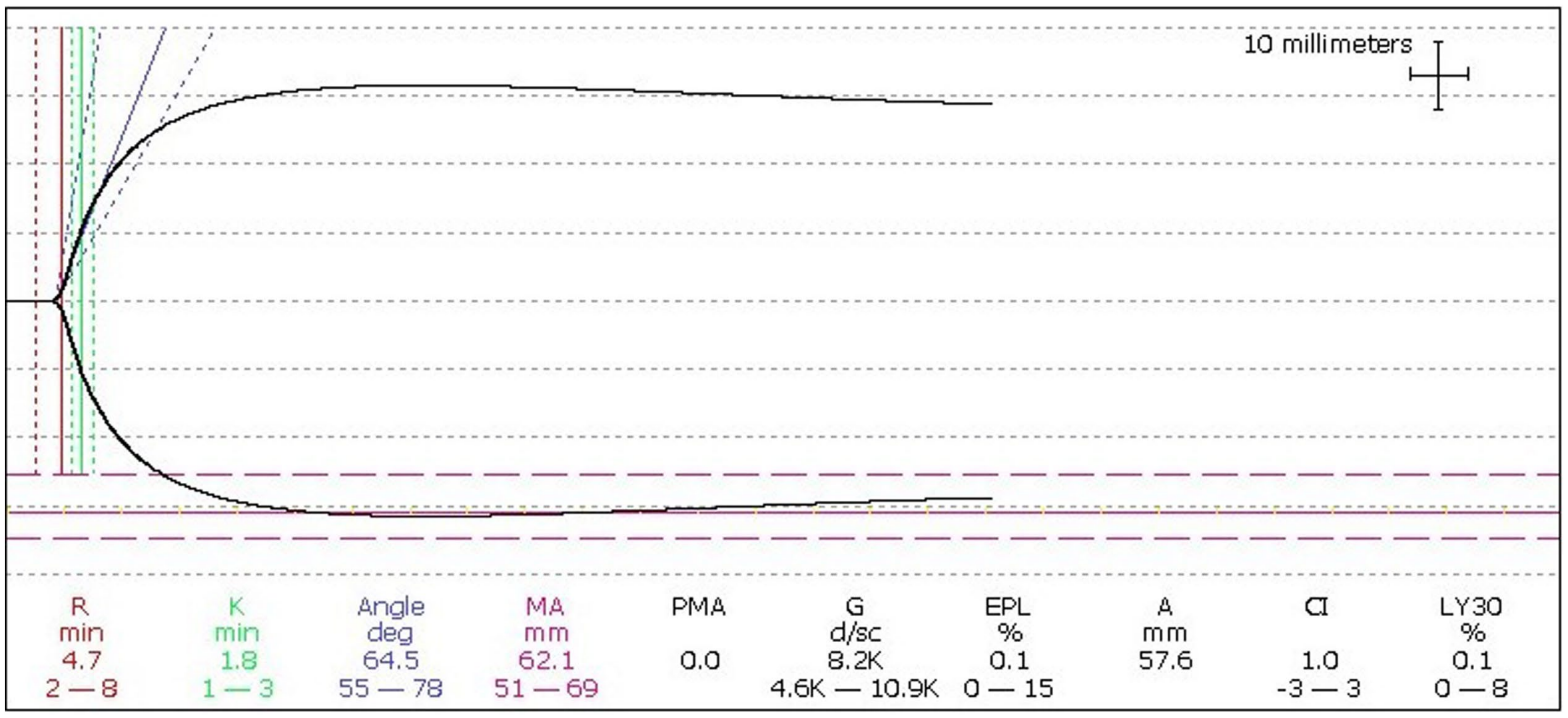
TEG following the administration of pdFVIII concentrate. [TEG—Thromboelastography, pd. FVIII—plasma-derived factor VIII, R—reaction time to initial clot formation (2–8 min), K—kinetics time from initial clot formation until reaching 20 mm in amplitude (1–3 min), α—alpha angle between the baseline at initial clot formation, and a tangent line that intersects the tracing curve (55–78), MA—maximum amplitude at peak clot strength (51–69), LY30—lysis at 30 min (0–8)].

**Figure 2:**
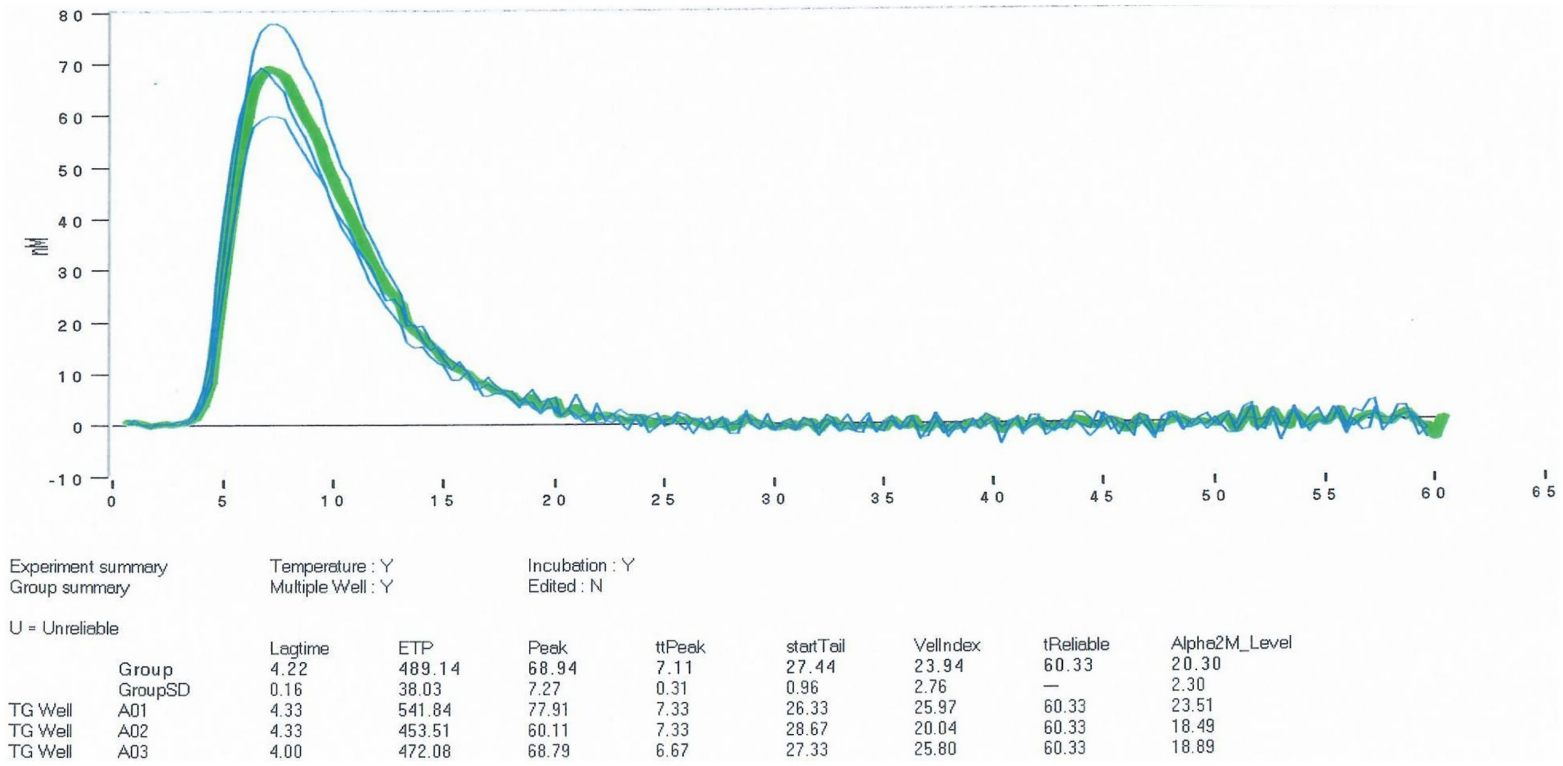
TGA following pdFVIII concentrate administration: [TGA—Thrombin Generation Assay, pdFVIII—plasma-derived factor VIII, ttPeak—Time to Peak (min), ETP—Endogenous Thrombin Potential (nM·min), VelIndex—Velocity Index (nM/min)].

One hour after administration, and considering the concomitant use of pro- and anticoagulant therapy, haemostatic efficacy was assessed using Thromboelastography (TEG) and Thrombin Generation Assay (TGA), showing improvements in r-time and maximum amplitude (MA) for clot formation (Figure 1), as well as in TGA parameters such as peak thrombin and Endogenous Thrombin Potential (ETP) (Figure 2). Plasma FVIII activity was 124%.

A full median sternotomy and pericardiotomy were performed. Extracorporeal circulation with cannulation for anterograde and retrograde cardioplegia to ensure myocardial protection via cooled crystalloid perfusion was initiated. Standard venting via the right superior pulmonary vein into the left ventricle was performed. After aortic clamping, a longitudinal aortotomy was performed extending to the non-coronary sinus, with retrograde and anterograde cardioplegia administered directly into the coronary ostia. On examination, a tricuspid aortic valve, prolapse of the right coronary cusp., and a dilated aortic ring were noted, resulting in severe aortic regurgitation. The aortic valve was excised and replaced with an Edwards Lifesciences Perimount biological prosthesis. A concomitant decision was made, and a reduction plasty of the ascending aorta was performed by excising two strips of approximately 1 cm width from the aorta, followed by aortorrhaphy with double surjet using Teflon tape and Prolene 4/0; the aorta was tubularly reduced to approximately 35 mm diameter, maintaining its symmetry. The aortic cross-clamp was removed, followed by purging the heart cavities and a hot shot. The heart resumed activity in sinus rhythm. Reperfusion and a gradual reduction in extracorporeal circulation, together with venous and arterial decannulation, were performed. Sternorrhaphy and closure in anatomical layers, with intradermal sutures, finalised the intervention. The patient was extubated after 14 h, and cardiopulmonary bypass (CPB) was weaned after 1 h and 20 min. The chest tube was removed on postoperative day (POD) 1, after 20 h. Total postoperative drainage was 740 mL.

Subsequently, pd. FVIII concentrate was administered every 8 h for the next 3 days, then every 12 h until discharge, with dose adjustments based on plasma FVIII activity and the patient’s clinical progress. We targeted FVIII peak levels of 100% pre-, intra- and postoperatively, then gradually reduced them to 30–80% for 4–14 days after surgery. The patient had an intensive care unit (ICU) stay of 62 h and a hospital stay of 13 days. Haemostasis was maintained with 64,000 IU of plasma-derived F VIII over 13 days, complemented by transfusion of one unit of packed red blood cells (PRBCs) and three units of fresh frozen plasma (FFP). Hypocoagulability was maintained with unfractionated heparin (UFH) during CPB, neutralised with protamine sulphate after 2 h, and then continued with fractionated heparin. Hyperfibrinolysis was controlled with tranexamic acid, and myocardial protection was ensured by cardioplegia perfusion with cooled crystalloid solutions. Pharmacological thromboprophylaxis was initiated on POD 2 with enoxaparin, 4,000 IU administered every 12 h. After discharge, he remained on continuous FVIII prophylaxis and on fractionated heparin for 3 months, then switched to aspirin for a further 3 months.

Strict haemostatic monitoring was carried out. Bleeding risk secondary to haemophilia, heparinisation, surgical trauma, extracorporeal circulation, hypothermia, and increased fibrinolysis could be mitigated only based on a rigorous laboratory investigation. Notably, before starting CPB, intravenous replacement therapy with a plasma-derived coagulation factor was initiated, with the rhythm and dosage carefully adjusted to the patient’s FVIII level and thrombin generation potential. Haemostatic control was challenging and was assessed using a standard coagulogram (APTT, PT, TT, fibrinogen, platelet count, D-dimers), FVIII activity and inhibitor levels, TEG, and TGA. During the first 3 days, these tests were performed 2–3 times per day; thereafter, they were performed daily. Long-term follow-up over the years showed a favourable outcome, with no bleeding or thrombotic events.

## Discussion

The *deficiency of coagulation* factors in haemophilia may provide a protective effect against thrombus formation, but it does not influence the mechanism of atherosclerotic development. Even severe haemophilia, defined by an FVIII level of <1%, with spontaneous, life-threatening bleeds, unfortunately, can not protect PwH against atherosclerosis and its associated cardiovascular diseases (2, 3). This explains why haemophiliacs are not protected against cardiovascular diseases; approximately 30% of PwH over the age of 60 with moderate/severe haemophilia are affected (4–6, 13, 14). As a result, haematologists are now confronted with a growing population of older PwH who develop age-related diseases, including ischaemic heart disease and degenerative valve disease, that require surgical intervention (14–18).

The extensive list of factors that expose individuals, including those without haemophilia, to complications such as excessive bleeding or thrombosis during invasive heart surgery is overwhelming (9, 19, 20).

For valvulopathies, new, less risky treatment modalities have been introduced. Transcatheter aortic valve implantation (TAVI) or replacement (TAVR) is now feasible and increasingly performed in the general population, including also people with haemophilia. These procedures, performed in most cases, are less invasive than open-heart surgery with cardiopulmonary bypass and are associated with lower rates of major haemorrhagic complications; they are increasingly being applied today to a larger population of bleeding-risk patients (8, 21). However, severe comorbidities, a markedly enlarged ascending aorta, an inadequate aortic annulus, and a patient-specific bicuspid aortic valve are contraindications to TAVI/TAVR. Consequently, in our patient, the indication for TAVI or TAVR was waived, and surgical intervention was performed in accordance with accepted guidelines, as in patients without haemophilia.

Generally, cardiac surgery with CPB is associated with:

- a complex coagulopathy involving both dilution and consumption, with platelet loss and a significant (35–49%) reduction in fibrinogen and other coagulation factors (II, V, X, XIII), due to blood flow over the large surface area of the tubing in the heart-lung machine and to volumetric correction and priming of the CPB circuit with crystalloid or colloid solutions.
- blood collection before surgery,
- reversal of autologous blood priming post-surgery,
- trauma of sternotomy, venesection, arterial and venous cannulation,
- hypotermia, acidosis, hypocalcaemia, these being only some of the procedure-related factors that disturb normal haemostasis (22).

There are also some additional challenging *risk factors related to medication*.

Generally, continuous infusion (CI) is advocated, but bolus administration is recognised as the “fallback” in numerous clinical settings, provided the trough level is not allowed to fall below 50% during the initial 72 h. Cardiac surgery presents a specific challenge known as the “dilution effect.” When the patient is connected to CPB, the pump prime comprising crystalloids and colloids, substantially dilutes circulating FVIII. Several Japanese case reports describe haemophiliacs undergoing cardiac surgery for degenerative valvular disease, with bolus injection used for its simplicity of administration in the ICU setting, resulting in successful outcomes. However, haemostatic security in aortic surgery depends more on trough-level management than on the delivery method. Therefore, continuous perfusion is preferred (10–12).

Regarding the type of coagulation product, the use of pdFVIII versus rFVIII is also discussed; the literature supports the use of pdFVIII in complex surgeries because it may reduce inhibitor formation compared with intensive rFVIII exposure during surgery. The vWF content in pdFVIII may offer an unquantified yet advantageous “side effect” in the context of CPB-induced coagulopathy (10, 23).

The usage of some drugs with an impact on haemostasis is another important subject:

- administration of epsilon-aminocaproic acid (EAC) or tranexamic acid, both antifibrinolytic agents, to prevent massive fibrinolysis,
- usage during CPB of UFH, followed by its neutralisation at the end of CPB with protamine sulphate (24).
- long-term (3-month) anticoagulation with low molecular weight heparin,
- usage of platelet anti-aggregants (aspirin/clopidogrel), all interfering with multiple steps of haemostasis (9, 12, 20, 22).

In this disrupted biological environment, where conflicting factors such as pro- and anticoagulants, pro- and anti-fibrinolytics, and platelet anti-aggregants are present, maintaining a balance of haemostatic factors is both challenging and essential. The medical actions intended to achieve a protective, safe, and secure outcome are consequently demanding and obligatory (8, 10, 11). This could only be based on accurate, continuous *laboratory monitoring*, which is a key requirement and a prerequisite for individualised replacement therapy. However, this monitoring is unfortunately difficult to perform. A routine panel of coagulation tests does not provide reliable results.

Heparin and its neutralising agent, protamine sulphate, interfere with APTT, FVIII assay, and FVIII inhibitor testing, leading to confounding effects that compromise test accuracy. Notably, assays based on fibrin formation (PT, aPTT) have limited clinical utility, as their values reflect the initiation phase of coagulation, during which only 2–5% of normal thrombin is sufficient for initiation. This could explain false-normal results in cases of coagulation deficits. Additionally, PT and aPTT are primarily sensitive only to severe hypofibrinogenaemia (<60–70 mg/dL) and are insensitive to FXIII deficiency or fibrinolysis (24, 25).

An additional characteristic to consider is their prolonged turn-around time; the delayed results may no longer accurately reflect the current coagulation status. Regarding monitoring FVIII activity, testing procedures can be unreliable. It is recognised that there is considerable clinical heterogeneity among patients with identical levels of factor deficiency; reports indicate that 10–15% of individuals with severe forms of haemophilia A (FVIII < 1%) exhibit a mild clinical phenotype. Furthermore, patients with non-severe haemophilia (NSH) may present with a severe phenotype. Consequently, reliance solely on FVIII concentration may not always provide sufficient insight (2, 3).

Thus far, two types of new bedside assays have been introduced over the past decade. One is based on thrombin generation, hereafter referred to as TGA or calibrated automated thrombin generation (CAT), and the other is based on measuring the viscoelastic properties of whole blood, known as TEG. CAT provides continuous quantification and evaluation of thrombin generation, with parameters including lag time, time to peak, and peak endogenous thrombin potential. The laboratory phenotype is concordant with the clinical phenotype, and TEG and CAT also reflect the dynamics of the coagulation process (26–30).

These complementary assays, TEG and CAT, are recommended to be used as:

- surrogate measures of the whole hemostatic efficacy,
- assessment of the clinical heterogeneity and phenotyping of the disease,
- giving information on clot stability and fibrinolysis, with
- information accessible point of care. The goals for the future are to obtain objective, reliable point-of-care laboratory outcomes by applying simple, inexpensive, reliable, informative data that correlate well with clinical outcomes (26, 27, 31, 32).

Throughout the patient’s entire care, including pre-, intra-, and post-operatively, we have considered all the aforementioned provocative impacts of haemophilia itself, the medication used, and monitoring control on the safety of the surgical intervention.

With the novel technological advances in comprehensive point-of-care coagulation tests, it was possible to assess the risk of blood loss and promptly identify other specific haemostatic defects, enabling timely correction with targeted treatment.

## Conclusion

Major cardiac surgery could be performed safely in our patient with severe haemophilia A. A multidisciplinary, experienced team approach under strict perioperative monitoring was indispensable in our case to achieve optimal outcomes.

## Data Availability

The raw data supporting the conclusions of this article will be made available by the authors upon reasonable request.

## References

[1] Makris M Hermans C. A golden age for Haemophilia treatment?. Haemophilia: the official journal of the world federation of. Haemophilia. (2018) 24:175–6. doi: 10.1111/hae.13411 29601683

[2] Oldenburg J. Optimal treatment strategies for haemophilia: achievements and limitations of current prophylactic regimens. Blood. (2015) 125:2038–44. doi: 10.1182/blood-2015-01-528414, 25712992

[3] Hart DP Kessler CM Aledort L. Re-personalisation and stratification of haemophilia care in an evolving treatment landscape. Haematology (Amsterdam, Netherlands). (2019) 24:737–41. doi: 10.1080/16078454.2019.1687798 31711380

[4] Mannucci PM Schutgens RE Santagostino E Mauser-Bunschoten EP. How I treat age-related morbidities in elderly persons with haemophilia. Blood. (2009) 114:5256–63. doi: 10.1182/blood-2009-07-215665, 19837978

[5] Miesbach W Alesci S Krekeler S Seifried E. Comorbidities and bleeding pattern in elderly haemophilia a patients. Haemophilia: the official journal of the world federation of. Haemophilia. (2009) 15:894–9. doi: 10.1111/j.1365-2516.2009.02030.x, 19473414

[6] Mannucci PM Mauser-Bunschoten EP. Cardiovascular disease in haemophilia patients: a contemporary issue. Haemophilia. (2010) 16:58–66. doi: 10.1111/j.1365-2516.2010.02262.x, 20586804

[7] Marcucci CE Schoettker P. Perioperative Hemostasis: Coagulation for Anesthesiologists. Springer: Springer-Verlag Berlin Heidelberg (2015).

[8] Franchini M Focosi D Mannucci PM. How we manage cardiovascular disease in patients with haemophilia. Haematologica. (2023) 108:1748–57. doi: 10.3324/haematol.2022.282407.wheat, 36700406 PMC10316236

[9] Shalabi A Kachel E Kogan A Sternik L Grosman-Rimon L Ben-Avi R . Cardiac surgery in patients with Haemophilia:is it safe? J Cardiothorac Surg. (2020) 15:76. doi: 10.1186/s13019-020-01123-0, 32384896 PMC7206692

[10] Kwak J Mazzeffi M Boggio LN Simpson ML Tanaka KA. Haemophilia: a review of perioperative management for cardiac surgery. J Cardiothorac Vasc Anesth. (2022) 36:246–57. doi: 10.1053/j.jvca.2020.09.118, 33082094

[11] Lin PS Yao YT. Perioperative Management of Haemophilia a Patients Undergoing Cardiac Surgery: a literature review of published cases. J Cardiothorac Vasc Anesth. (2021) 35:1341–50. doi: 10.1053/j.jvca.2020.06.074, 32723585

[12] Pagano D Milojevic M Meesters MI Benedetto U Bolliger D von Heymann C . 2017 EACTS/EACTA guidelines on patient blood management for adult cardiac surgery. Eur J Cardiothorac Surg. (2018) 53:79–111. doi: 10.1093/ejcts/ezx325, 29029100

[13] Coppola A Tagliaferri A Franchini M. The Management of Cardiovascular Diseases in patients with hemophilia. Semin Thromb Hemost. (2010) 36:91–102. doi: 10.1055/s-0030-1248728, 20391300

[14] Ferraris VA Boral LI Cohen AJ Smyth SS White GC 2nd. Consensus review of the treatment of cardiovascular disease in people with haemophilia a and B. Cardiol Rev. (2015) 23:53–68. doi: 10.1097/CRD.0000000000000045, 25436468 PMC4323575

[15] Xu H Henry D Li C Zhao H Yang Y. Peri-cardiac surgery coagulation management in a severe hemophilia a patient: a case report. Medicine (Baltimore). (2019) 98:e15897. doi: 10.1097/MD.0000000000015897, 31192923 PMC6587658

[16] Odonkor P Srinivas A Strauss E Williams B Mazzeffi M Tanaka KA. Perioperative coagulation Management of a Haemophilia a Patient during Cardiac Surgery. Semin Cardiothorac Vasc Anesth. (2017) 21:312–20. doi: 10.1177/1089253217702747, 28388863

[17] Davidson S. State of the art - how I manage coagulopathy in cardiac surgery patients. Br J Haematol. (2014) 164:779–89. doi: 10.1111/bjh.12746, 24450971

[18] Podolak-Dawidziak M Biernat M Bator M Urbaniak-Kujda D Zoń A Wróbel T. Multifactorial assessment of cardiovascular risk in patients with severe haemophilia from the lower Silesia region in Poland. Acta Haematol Pol. (2025) 56:316–25. doi: 10.5603/ahp.106915

[19] Mistry T Dogra N Chauhan K Shahani J. Perioperative considerations in a patient with Haemophilia a: a case report and review of literature. Anesth Essays Res. (2017) 11:243–5. doi: 10.4103/0259-1162.181432, 28298793 PMC5341657

[20] Meesters MI von Heymann C. Optimizing perioperative blood and coagulation management during cardiac surgery. Anesthesiol Clin. (2019) 37:713–28. doi: 10.1016/j.anclin.2019.08.006, 31677687

[21] Güney MC Karaduman BD Ayhan H Kele T Bozkurt E. Transcatheter aortic valve implantation in bicuspid aortic valve patients with coagulation factor 7 and 11 deficiency and atrial fibrillation. Anatol J Cardiol. (2023) 27:173–5. doi: 10.14744/AnatolJCardiol.2022.2842, 36856598 PMC9995554

[22] Bhave P McGiffin D Shaw J Walsh M McCarthy P Tran H . Guide to performing cardiac surgery in patients with hereditary bleeding disorders. J Card Surg. (2015) 30:61–9. doi: 10.1111/jocs.12464, 25345869

[23] Bolliger D Vandyck K Tanaka KA. Management of Patients with Haemophilia Undergoing Cardiac Surgery. J Cardiothorac Vasc Anesth. (2022) 36:539–41. doi: 10.1053/j.jvca.2021.11.022, 34933808

[24] Khandelwal A Phua CW Chaudhry HR Tsui H Rivard GE Teitel JM . Confounding effect of therapeutic protamine and heparin levels on routine and special coagulation testing. Blood Coagul Fibrinolysis. (2020) 31:60–4. doi: 10.1097/MBC.0000000000000882, 31904611

[25] Strauss ER Mazzeffi MA Williams B Key NS Tanaka KA. Perioperative management of rare coagulation factor deficiency states in cardiac surgery. Br J Anaesth. (2017) 119:354–68. doi: 10.1093/bja/aex198, 28969316

[26] Șerban M Poenaru D Cernat L Savescu D Pătrașcu J Schramm W . Global clotting assays - monitoring the effect of bypassing agents in haemophilia patients with inhibitors. Rev Romana Med Lab. (2017) 25:135–43. doi: 10.1515/rrlm-2017-0011

[27] Melen B Uscatescu V Gheorghe G Chiriac E Ciobanu C Orban H . Thromboelastography in pre-surgery monitoring in Haemophilia a with high inhibitor titer: case report and literature review. Rev Romana Med Lab. (2020) 28:217–24. doi: 10.2478/rrlm-2020-0021

[28] Tanaka KA Henderson RA Strauss ER. Evolution of viscoelastic coagulation testing. Expert Rev Hematol. (2020) 13:697–707. doi: 10.1080/17474086.2020.1758929, 32310702

[29] Nogami K. The utility of thromboelastography in inherited and acquired bleeding disorders. Br J Haematol. (2016) 174:503–14. doi: 10.1111/bjh.14148, 27264484

[30] Chitlur M Rivard GE Lillicrap D Mann K Shima M Young G . Recommendations for performing thromboelastography/thromboelastometry in haemophilia: communication from the SSC of the ISTH. J Thromb Haemost. (2014) 12:103–6. doi: 10.1111/jth.12458, 24261669

[31] Tanaka KA Bolliger D Vadlamudi R Nimmo A. Rotational thromboelastometry (ROTEM)-based coagulation management in cardiac surgery and major trauma. J Cardiothorac Vasc Anesth. (2012) 26:1083–93. doi: 10.1053/j.jvca.2012.06.015, 22863406

[32] Young G Sørensen B Dargaud Y Negrier C Brummel-Ziedins K Key NS. Thrombin generation and whole-blood viscoelastic assays in the management of haemophilia: current state of the art and future perspectives. Blood. (2013) 121:1944–50. doi: 10.1182/blood-2012-08-378935, 23319573 PMC3645054

